# Comparisons of Methods for Mucus Sampling and Mucin Semi-Quantification on Barramundi (*Lates calcarifer)* and Atlantic Salmon (*Salmo salar*) Epithelial Sites

**DOI:** 10.1007/s10126-025-10515-z

**Published:** 2025-09-24

**Authors:** Kyung Min Lee, John Benktander, James W. Wynne, Joel Slinger, Richard S. Taylor, Sara K. Lindén

**Affiliations:** 1 Department of Medical Biochemistry and Cell Biology, Institute of Biomedicine, Sahlgrenska Academy, University of Gothenburg, Medicinaregatan 9C, Box 440, 405 30 Gothenburg, Sweden; 2 CSIRO Agriculture and Food, Hobart, TAS 7001 Australia; 3 CSIRO Agriculture and Food, Bribie Island Research Centre, Woorim, Welsby 4507 Australia

**Keywords:** Mucus sampling, Mucus semi-quantification, Glycan, Atlantic salmon, Barramundi

## Abstract

Fish epithelial surfaces are covered by a mucus layer. The highly glycosylated proteins called mucins are a main component of the mucus, which also contains a range of antibacterial enzymes, proteins, and peptides of importance for its protective properties. Here, we compared the practicality and yield of mucus harvesting from barramundi and Atlantic salmon epithelial sites using glass slide, swab, Super·SAL™ and whole tissue extract. We also compared the feasibility of using the orcinol assay, a glycan-on-membrane assay, and absorbance at 230 nm in combination with standard curves of pig gastric mucin to estimate the mucin concentration. Glycomics demonstrated that non-amine hexose content differed more between fish and tissues than terminal monosaccharides with cis-hydroxy groups, and that non-mucin molecules had a major impact on the A230-based results, making the glycan-on-membrane assay the most versatile method for estimating mucin concentration. We conclude that the most versatile tool for mucus harvesting was swabs, allowing for sufficient amounts of sample to be harvested with relative ease and low levels of contamination from the oral cavity, gill, skin, and intestine. Furthermore, the glycan-on-membrane assay was useful for measuring mucus concentration, and it was beneficial to estimate both sample concentration and purity by comparing samples at relatively similar concentrations.

## Introduction

Fish epithelial surfaces are covered by mucus, a gel-like substance containing antibacterial enzymes, proteins, and water, which plays an important role in preventing the invasion of pathogens (Linden et al. 2008; Dash et al. 2018). The main component of mucus is the highly glycosylated proteins called mucins, which are responsible for forming a viscous texture. The majority of mucins are composed of carbohydrates (glycans), which constitute over 70% of the total composition of the mucins (Linden et al. 2008). The mucin glycans directly bind to pathogens and also regulate other factors related to pathogens, such as quorum sensing, growth, virulence, attachment to the host epithelial cells, and composition of the microbiota (Willing et al. 2010; Padra et al. 2014; Skoog et al. 2017; Lima et al. 2021; Wu et al. 2023; Loibman et al. 2024). Over the past decade, there has been a notable increase in research in the field of fish mucus, including detailed studies on glycans and antimicrobial properties (Jin et al. 2015; Venkatakrishnan et al. 2019; Thomsson et al. 2022, 2024; Bachar-Wikström et al. 2023; Díaz-Puertas et al. 2023; Hussain and Sachan 2023).

Atlantic salmon (*Salmo salar*) is one of the most economically important fish species in aquaculture, and its cultivation and production are increasing worldwide with a high demand for consumption (Houston and Macqueen 2019). In 2020, farmed Atlantic salmon constituted the largest proportion (71.4%) of total salmon production, representing a markedly increased value in comparison with the production in 2000 (47.3%) (Pandey et al. 2023). In 2023, its production in aquaculture reached approximately 2.9 million tonnes (Knudson and Peterson 2001). The main producers are Norway, Chile, Scotland, Canada, the Faroe Islands, and Australia (McIntosh et al. 2022).

Barramundi (*Lates Calcarifer*), also known as Asian seabass, is a euryhaline species that is native to the Indo-Pacific regions and is widely distributed in the region from South Asia, Papua New Guinea, to Northern Australia (FAO 2021). This species demonstrates substantial environmental adaptability across aquaculture systems, is tolerant of high stocking densities, and readily accepts formulated aquafeeds (Boonyaratpalin 1997). Furthermore, barramundi can reach commercial size within a relatively short period (6 months–2 years) and is regarded as a highly preferred product by consumers, which has contributed to a steady increase in its cultivation (FAO 2021).

As farmed production of these two key fish species continues to expand, the prevalence of fish diseases, including bacterial, parasitic, and viral infections, has become a significant concern for the aquaculture industry (Sajid et al. 2024; Yue and Guo 2025). Given that most fish pathogens initiate infection through a mucosal portal of entry (Gomez et al. 2013), it is imperative to investigate the mucus-based defences as the mucus is the first barrier pathogens encounter. To achieve the objective of analysing mucus or mucus components, including the large mucin glycoproteins that make up the mucus gel, mucus from the target area or organ is collected. The commonly used methods to harvest mucus can broadly be classified into scraping, absorption, and bagging (Cunha et al. 2024).

The scraping method employs a variety of instruments, including cell scrapers, spatulas, or glass slides to scrape mucus from the surface of the target fish organ. This is the most commonly employed method for processing various fish samples, including skin, gill, and intestinal tissue. The collection procedure is relatively brief and may yield a higher mucus volume and protein concentration compared to the absorption method (Cunha et al. 2024). However, the scraping process exerts a slight pressure on the surface of fish skin, which can result in the secretion of additional mucus and the dislodgement of scales (Khorshidi et al. 2021; Cunha et al. 2024). Furthermore, it is recommended that the process be completed within a few minutes to avoid the degradation of mucus metabolites (Firmino et al. 2021).

The bagging method places the fish in a plastic bag, flask, or beaker with solution, and then the fish is massaged to facilitate the release of mucus. This method is mainly employed for the collection of skin mucus, but massaging may easily contaminate the sample with faeces or blood. The mucus collected using this method is diluted with the solution, resulting in samples that contain other substances from the solution. Thus, a subsequent centrifugation step is required to remove contaminants following collection. So far, despite numerous studies that utilised this method in fish samples, the solution and the requisite time have not been optimised, with considerable variation between studies (Cunha et al. 2024). Furthermore, there is a risk that the fish suffer during sampling by bagging, requiring fish to be euthanised or deeply anaesthetised prior to sampling.

The absorption method is also mainly utilised for the collection of skin and gill mucus. In this method, a suitable absorbent material, such as cotton, medical wipes, or soaking papers, are applied to the body surface and then removed once saturation is achieved. This method provides a less-contaminated mucus sample than the scraping and bagging method, and the centrifugation step can be omitted (Cunha et al. 2024). However, the protein concentrations and volumes obtained are typically lower than those obtained through scraping and wiping methods (Tartor et al. 2020; Bachar-Wikström et al.^2023^), although, to our knowledge, direct comparisons have not yet been conducted. Collecting samples via swab represents a somewhat intermediate approach between scraping and absorption methods, as it is less invasive than conventional scraping techniques while still involving the movement of the swab across the body surface.

When analysing mucus/mucins, it is often necessary to know the amount and concentration of the material, a factor that is especially important when comparing mucus from different experimental groups to accurately compare impacts such as feed components, infection, temperature or other treatment, or to compare between different species/epithelia. The proportion of water contained within a collected mucus sample can change rapidly after lifting the fish out of the water, making the option of simply weighing the native mucus unreliable for comparisons. Determining the concentration of mucins is complicated, as they are glycoconjugates, with a high proportion of glycan. One option to determine the amount of mucin in a sample is by freeze-drying and weighing, however, loss of material due to incomplete mucin solubility after freeze-drying is a common problem, which may lead to selective loss of certain mucin glycoforms. Also, non-mucin protein and lipid contaminants may contribute to the weight. Thus, mucins recovered through freeze-drying may not be fully representative of the original mucins. There is also a loss of precision when freeze-drying and weighing small sample quantities. Therefore, it is often desirable to measure the mucin concentration in solution. Studies that include several study groups also often result in a large number of samples, requiring methods that are relatively fast to be feasible.

Given that glycans constitute the majority of mucin molecules and are critical to their functional properties, quantifying mucins based on glycan content is often the most appropriate approach. Since the mucin molecules consist mainly of glycan, the signal obtained by protein-based assays is relatively low, and contaminants from non-mucin proteins can contribute disproportionally to the apparent mucin content. The orcinol assay is commonly used for determining mucin concentration based on glycan content and is a microtitre plate-based assay using equipment available in most laboratories. It is a method that detects carbohydrates based on a colour reaction with orcinol and sulphuric acid after heating. The sulphuric acid in combination with heating breaks down the glycans into monosaccharides. However, the orcinol assay detects neutral sugars, such as hexoses, while amino sugars do not result in a colour reaction (Brückner 1955; Group 1987). Another method commonly used for determining mucin concentration is the glycan method. This method detects vicinal diols in cis-formation, and a signal is obtained for the most abundant terminal monosaccharides, including sialic acids, galactose (Gal), *N*-acetylgalactosamine (GalNAc), mannose (Man), and fucose (Fuc) in their common forms (Bayer et al. 1988). This method is commonly used in its microtiter-based format to determine the concentration of pure mucins (Padra et al. 2019; Quintana-Hayashi et al. 2021). However, when using mixed samples, such as mucus, which also contain non-mucin proteins, the assay needs to be performed on a membrane instead, as non-mucin proteins normally coat microtitre plates faster than mucins. Absorbance is a third method for estimating concentration, which can also be combined with wavelength scans to estimate the purity of the sample.

Although the methodology of mucus sampling in fish has not been extensively explored, several studies have documented that the results of mucus analyses, including proteome, metabolome, and immune molecule assessment, can vary depending on the sampling methods (Ivanova et al. 2018; Fæste et al. 2020; Tartor et al. 2020). In the present study, mucus samples were collected from barramundi and Atlantic salmon mucosal surfaces using cotton swab, glass slide, and Super·SAL™. The orcinol and glycan-on-membrane assay were used to determine the concentration and yield of the samples. We also investigated the option of using A230 to estimate concentration and yield. Advantages and limitations of using these different methods are also discussed.

## Material and Methods

### Mucin Isolation Buffer

A phenylmethanesulfonyl fluoride (PMSF) stock solution was made up with 200 mM PMSF, 1 g in 28 mL of ethanol, then stored at 4 °C. Next, 0.1 mM sodium dihydrogen phosphate monohydrate (MW 137.99 g) was made to 10 mM by adding 0.69 g in 500 mL dH_2_0 and the pH was set to 6.5. Immediately prior to fish sampling, 25 µL of PMSF stock solution was added to 500 mL of 10 mM sodium dihydrogen phosphate. The mucin isolation buffer was kept on ice throughout sampling.

### Barramundi and Mucus Sampling

Barramundi were sourced from a brackish water commercial farm in Northern Australia (salinity 8.5 psu, temperature 29.6 °C). Mucosal surfaces were opportunistically sampled during a scheduled fish health check. Four fish (2030 ± 385 g) were captured from two earthen ponds (approximately 70 × 80 m each) using a small seine net and transferred by dip net to 150 mg∙L^−1^ anaesthetic (Aqui-S, Lower Hutt, New Zealand) for rapid individual euthanasia prior to sampling. To remove excess water and prevent contamination of the skin, each fish was initially suspended by grasping the lower jaw, allowing excess water to drip off until viscous mucus was observed to hang from the caudal fin margin (approximately 1 min).

**Fig. 1:**
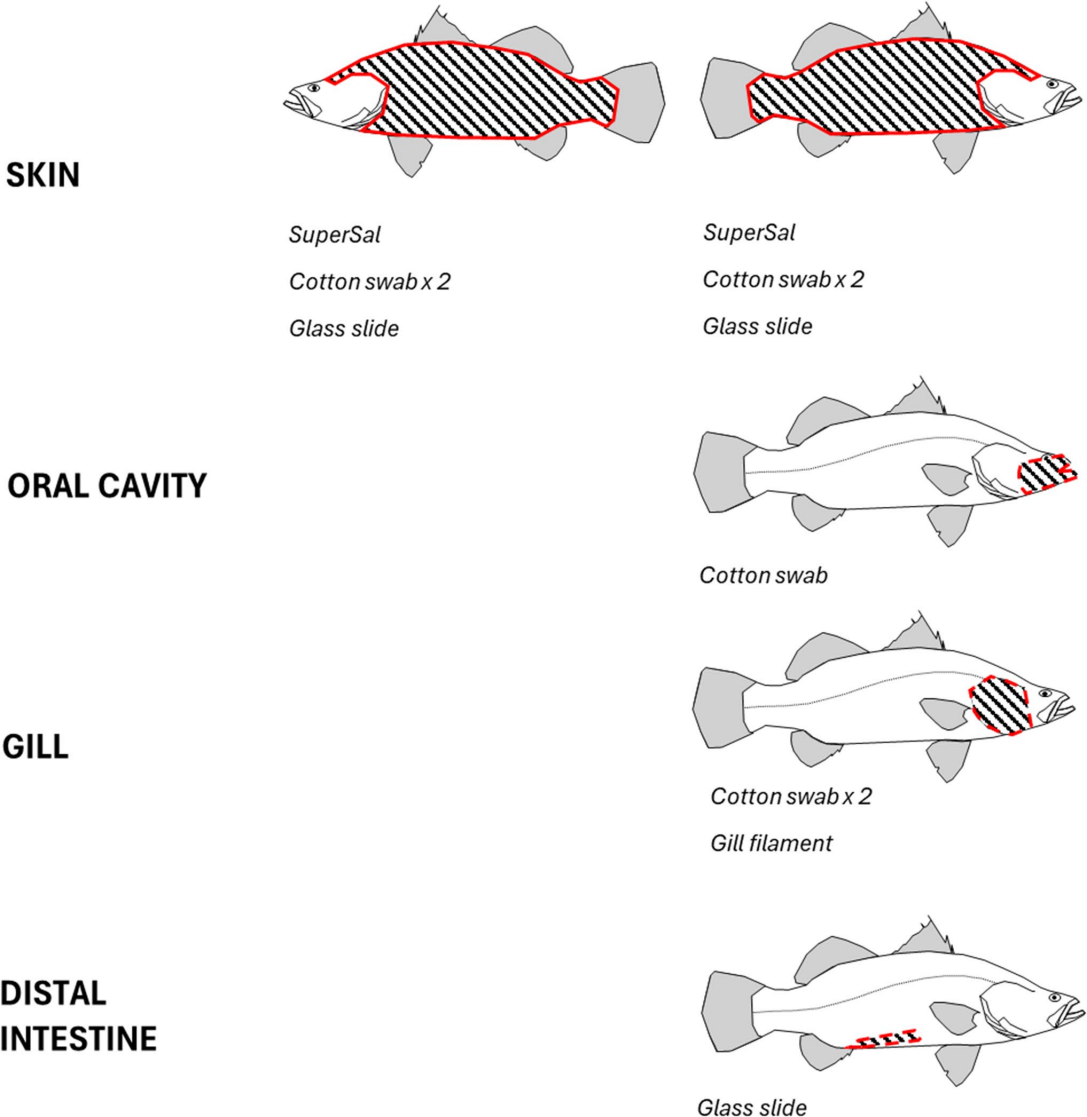
Mucosal sampling methods. Fish skin was sampled using Super·SAL™, cotton swab or glass slide. Gill was sampled using cotton swab and by taking a small piece of gill filament tissue. Oral cavity and distal intestine were sampled using cotton swab for barramundi only

As shown in Fig. 1, the left side (method (ii), (iii)) or both left and right sides (method (i)) of the body surface were then sampled using three collection methods: (i) a Super·SAL™ Oral Fluid Collection Device (Oasis Diagnostics, Vancouver, WA, USA), consisting of an absorbent pad held in a compression tube (Khurshid et al. 2016). The pad was drawn along the anterior dorsal surface until the volume adequacy indicator changed colour. The sample was then expressed into a 2-mL screw cap tube; (ii) a pair of sterile cotton swabs (Westlab, Australia) with snappable polystyrene sticks was wiped and rotated along the dorsal posterior surface until fully soaked. The swab heads were then snapped off into a 2-mL screw cap tube; (iii) a sterile glass microscope slide was scraped edge first along the ventral surface, and the collected mucus was guided into a 2-mL screw cap tube. Each sample from the left side was snap frozen on dry ice for further analysis.

The oral cavity was sprayed with mucin isolation buffer and sampled using a cotton swab, which was then placed into a 2-mL sample tube and immediately snap frozen. Next, the operculum was lifted and buffer sprinkled onto the gill surfaces prior to cotton swabbing all four holobranchs of the first and second right gill arches (R1 and R2) using two swabs. A small sample of gill filament from the second left gill (L2) was then aseptically removed and snap frozen in a 2-mL tube.

Finally, a section of distal hindgut was excised and then opened with a sterile scalpel. Using a sterile spatula, digesta was gently pushed aside and the exposed surface was sprinkled with mucin isolation buffer before sampling intestinal mucus using a sterile glass slide. The collected mucus was then transferred into a 2-mL sample tube and snap frozen.

### Atlantic Salmon and Mucus Sampling

Atlantic salmon were originally imported into Australia from the River Philip (Nova Scotia, Canada) and have been commercially farmed in Tasmania since the mid-1980s. Salmon used in this study were transferred as fry from a commercial hatchery in Tasmania to the Bribie Island Research Centre (Queensland) in March 2022 and reared in a freshwater recirculation aquaculture system. The fish were smoltified and transferred to a seawater tank in August 2023. Four fish (2241 ± 403 g) were sampled (13.1 °C, 33.2 psu) using the same techniques as those described above. Fish samples obtained were scavenged from fish which had been euthanised in 150 mg∙L^−1^ Aqui-S during routine grading practices. Skin mucus samples were similarly taken from barramundi using the Super·SAL™ and glass slide methods, and samples were snap frozen. Finally, after mucin isolation buffer had been applied, gill mucus (swab and gill filament) samples were taken. No Atlantic salmon hindgut samples were gathered.

### Transfer Samples to Sweden

Samples were dispatched in 15 kg of dry ice from Australia to Sweden. Dry ice levels were managed during transport in order to ensure that sample integrity was maintained.

### Reference Mucin Samples

Four reference mucins were used: pig gastric mucin (PGM type II, Sigma-Aldrich) and mucins isolated from Atlantic salmon gill, skin, and distal intestine isolated using isopycnic GuHCl/CsCl density gradient centrifugation. This isolation method separates mucins from DNA (which has a higher density than mucins) as well as from less glycosylated proteins and lipids (which have a lower density than mucins) and was performed as previously described (Padra et al. 2014). DNA from Atlantic salmon skin, separated from mucins using density gradient centrifugation, was also included as a control.

### Mucin Extracts

To obtain mucus extracts containing mucins and additional soluble mucus components from barramundi and Atlantic salmon mucus samples, the samples were suspended in fivefold the sample volume in extraction buffer (6 M guanidine hydrochloride [GuHCl], 5 mM EDTA, 10 mM sodium phosphate buffer, 5 mM N-ethylmaleimide, 0.1 mM PMSF, and pH 6.5) and gently shaken overnight at 4 °C. Tissues (gills) were homogenised using a Dounce homogeniser in the extraction buffer before the overnight shaking. The samples were centrifuged at 4 °C for 80 min at 3900 × g, and then the supernatant was transferred to a new tube and stored at − 20 °C until use.

### O-glycan Analysis by Liquid-Chromatography–Mass Spectrometry (LC–MS)

O-glycans were released from fish mucins (approximately 100 μg) by reductive β-elimination and analysed by LC–MS as previously described (Benktander et al. 2019). The glycans from barramundi and Atlantic salmon can be found at https://unicarb-dr.glycosmos.org/references/GPST000507.0/f_0000045258, https://unicarb-dr.glycosmos.org/references/392, and 10.1021/acs.jproteome.5b00232. To calculate the average number of hexoses (i.e. glucose, galactose, mannose, and fucose) in the glycan structures, i.e. due to their detection by the orcinol-sulphuric acid assay, the relative abundance (%) of the individual glycans was multiplied by their hexose composition. The sum of the multiplied glycans was divided by 100 to obtain the average number of hexose monosaccharides on the structures. By calculating this, the difference in hexose content between different mucus samples can be compensated for when using the orcinol-sulphuric acid assay to determine mucin concentration.

To calculate the structures detectable with the glycan-on-membrane assay, the average number of terminal sialic acids, galactose, *N*-acetylgalactosamine, mannose, and fucose was calculated. This was done by multiplying the number of detectable monosaccharides by the relative abundance of the structures (%). The summed-up multiplied detectable monosaccharides from the glycans were divided by 100 to obtain the average detectable monosaccharides of the glycans.

### Orcinol Assay

Extracted mucins (10 µL) were mixed with 100 µL of orcinol solution (80 mg of orcinol monohydrate in 5 mL H_2_O was transferred to 37.5 mL of 60% sulphuric acid) in duplicate in a 96-well plate (Thermo Scientific) and incubated at 95 °C for 30 min. After cooling to room temperature for several minutes, the absorbance was measured in a CLARIOstar Plus Microplate Reader (BMG Labtech) at 405 nm. The concentration of mucin was calculated based on the standard curve generated using PGM in guanidinium hydrochloride (0.125–2 mg/mL).

### Glycan-on-Membrane Assay

A 10- and 20-fold diluted extracted mucins (100 µL) were loaded in duplicate onto an Immobilon-FL PVDF membrane (Millipore) using a slot blot apparatus (Bio-Dot Apparatus, Bio-Rad). Vacuum was applied to attach the mucins to the membrane, and the membrane was air-dried. The membrane was pre-wetted briefly in absolute methanol, rinsed with water and subsequently incubated in PBS containing 0.05% Tween 20 (PBS-T) for 10 min. The membrane was blocked with 1% BSA in PBS-T for 30 min, and then incubated with 10 mM sodium periodate solution for 30 min. The membrane was washed three times for 5 min with PBS-T and incubated with 3 µg/mL of biocytin hydrazide (Invitrogen) for 1 h. After washing three times, the membrane was incubated with streptavidin-labelled IRDye 800 (LICOR, Biosciences) diluted 1:5000 in PBS containing 0.01% SDS and 0.1% Tween 20 for 30 min. Following final washing, the membranes were visualised with an Odyssey CLx infrared imaging system (LI-COR, Biosciences). The concentration of mucins was calculated based on the standard curve generated using PGM in extraction buffer (0.05–0.75 mg/mL).

### Wavelength Scan of Mucin Samples

Absorbance was measured using a NanoDrop 2000 spectrophotometer (Thermo Scientific). The extraction buffer was used as a blank in the instrument. The mucin samples were measured at 230, 260, and 280 nm as well as full scans over the 220–350 nm area. The concentration of mucin was calculated based on the A230 nm standard curve generated using PGM in guanidinium hydrochloride (0.125–2 mg/mL).

## Results and Discussion

### Swabs were the Easiest and Most Versatile Tool for Sampling Mucus, Allowing Rapid and Low-Cost Mucus Collection from Skin, Mouth, and Gill with Minimal Contamination

The various scraping and swabbing methods utilised in this study of barramundi and Atlantic salmon skin, gills, mouth and hindgut were representative of common rapid mucosal sampling methods for field or laboratory use. We did not include the plastic bag massage technique as we considered this to be unsuitable for large (> 500 g) fish. Also, the bag technique could not be utilised without affecting other collection methods. Once excess water had been allowed to drip from the fish and mucosal strands were forming on the tail, swabbing could be done on both sides while the fish was suspended. Swabbing with cotton swabs was a rapid and low-cost technique. Since the swabs utilised were small (head size 15 × 5 mm), skin and gills were sampled with paired swabs. These small swabs were easy to use for skin, mouth and gill sampling with minimal contamination, as determined by visual inspection. In comparison, Super·SAL™ is a universal sampler for biological fluids which has been used with cows, horses, pigs, cats, non-human primates, dogs, and humans to sample hormones, bacteria, viruses, certain drug molecules, and proteins (Bellagambi et al. 2020). On > 2 kg fish, this system required little pressure to absorb skin mucus and provided clean samples of > 1.0 mL. However, the Super·SAL™ pads are too large to use for mouth or gill sampling and cost approximately 50 times as much as two swabs. Finally, scraping the skin using a glass microscope slide was a rapid collection method which tended to provide a ‘thicker’ or inconsistent sample indicative of skin damage. Slides can easily remove scales and cause bleeding from the scale bed. Although our researchers were able to easily lift the gathered mucus, transferring the sample into 2-mL tubes was more difficult than for swabs or Super·SAL™.

### The Non-Amine Hexose Content of Fish Mucins Differs Considerably Between Species and Epithelia, Which has to be Taken into Account when the Orcinol Assay is Used to Determine Concentration

The orcinol assay is commonly used for determining mucin concentration. One often forgotten complication of this assay is that the signal obtained is based on hexose content, i.e. sialic acids and amino-sugars, which dominate the glycan epitopes of mucins from some fish epithelia, do not contribute to the signal (Benktander et al. 2019). This factor may be less of an issue when comparing mucins from the same species and epithelial site but is likely to have a major effect when comparing mucins across different species or epithelial sites. To investigate how large this effect is, we compared the median number of non-amine hexoses per glycan on mucins from a range of epithelia from Atlantic salmon, barramundi and PGM and found that they differed. The mean number of non-amine hexoses per mucin glycan was lowest in Atlantic salmon skin (mean: 0.25) and barramundi oral cavity (mean: 0.28), followed by barramundi skin (0.38), barramundi gill (0.56), Atlantic salmon gill (0.78), and barramundi intestine (1.2) (Table 1). Notably, the mean number of non-amine hexoses per glycan on PGM (often used for making standard curves) is 1.90, e.g. 6.7-fold higher than for barramundi oral cavity and 7.6-fold higher than Atlantic salmon skin mucus, since the latter are mainly composed of hexosamines and sialic acids (Table 1).

**Table 1:** O-glycan size and number, composition, and factors for compensation of orcinol and glycan-on-membrane assay

Species	Tissue	Size: Average (SEM) number of monosaccharides	Average (SEM) Number of non-amine hexoses	Average (SEM) number of terminal cis-hydroxy groups	Multiplication factor for orcinol assay compensated with PGM	Multiplication factor for glycan-on-membrane assay compensated with PGM
Atlantic salmon	Gill	3.4 (0.095)	0.780 (0.088)	1.49 (0.041)	2.4	0.86
	Skin	2.4 (0.029)	0.249 (0.015)	1.10 (0.0089)	7.6	1.2
Barramundi	Gill	6.0 (0.47)	0.563 (0.054)	1.53 (0.076)	3.4	0.83
	Skin	5.5 (0.59)	0.375 (0.063)	1.40 (0.086)	5.1	0.91
	Oral	5.1 (0.83)	0.282 (0.047)	1.27 (0.085)	6.7	1.0
	Intestine	5.5 (0.11)	1.24 (0.11)	1.76 (0.072)	1.5	0.73
PGM		4.2	1.90	1.28		

### The Content of Terminal Monosaccharides with Cis-Hydroxy Groups is Relatively Similar Between Species, Epithelia and Individuals

A glycan-on-membrane assay was described already in 1988 (Bayer et al. 1988), which we adapted with a different detection system. The common monosaccharides that contribute towards the signal are sialic acid, Gal, GalNAc, Man, and Fuc in their common forms. However, due to the linkages between the monosaccharides, most of these cis-hydroxide groups are blocked, therefore generally only the terminal monosaccharides react with the biocytin hydrazide. The mean terminal cis-hydroxy group content was calculated for the mucins and found to be relatively similar between samples: Atlantic salmon skin mucin glycans had the lowest content (mean: 1.1) and barramundi intestinal mucin glycans the highest (1.8) and the remainder in between (Table 1). Thus, the difference between the highest and lowest was only 1.6-fold. Furthermore, PGM had a content of monosaccharides eliciting a signal similar to the fish mucins (1.3) (Table 1). Thus, the compensation factors needed to estimate the mucin concentration are much lower and differ less between epithelia for the glycan-on-membrane assay (0.73–1.2) than for the orcinol assay (1.6–7.6, Table 1).

### Mucus Was Obtained Using Most Harvesting Methods and Epithelial Surfaces, Albeit with Varying Yields

**Fig. 2:**
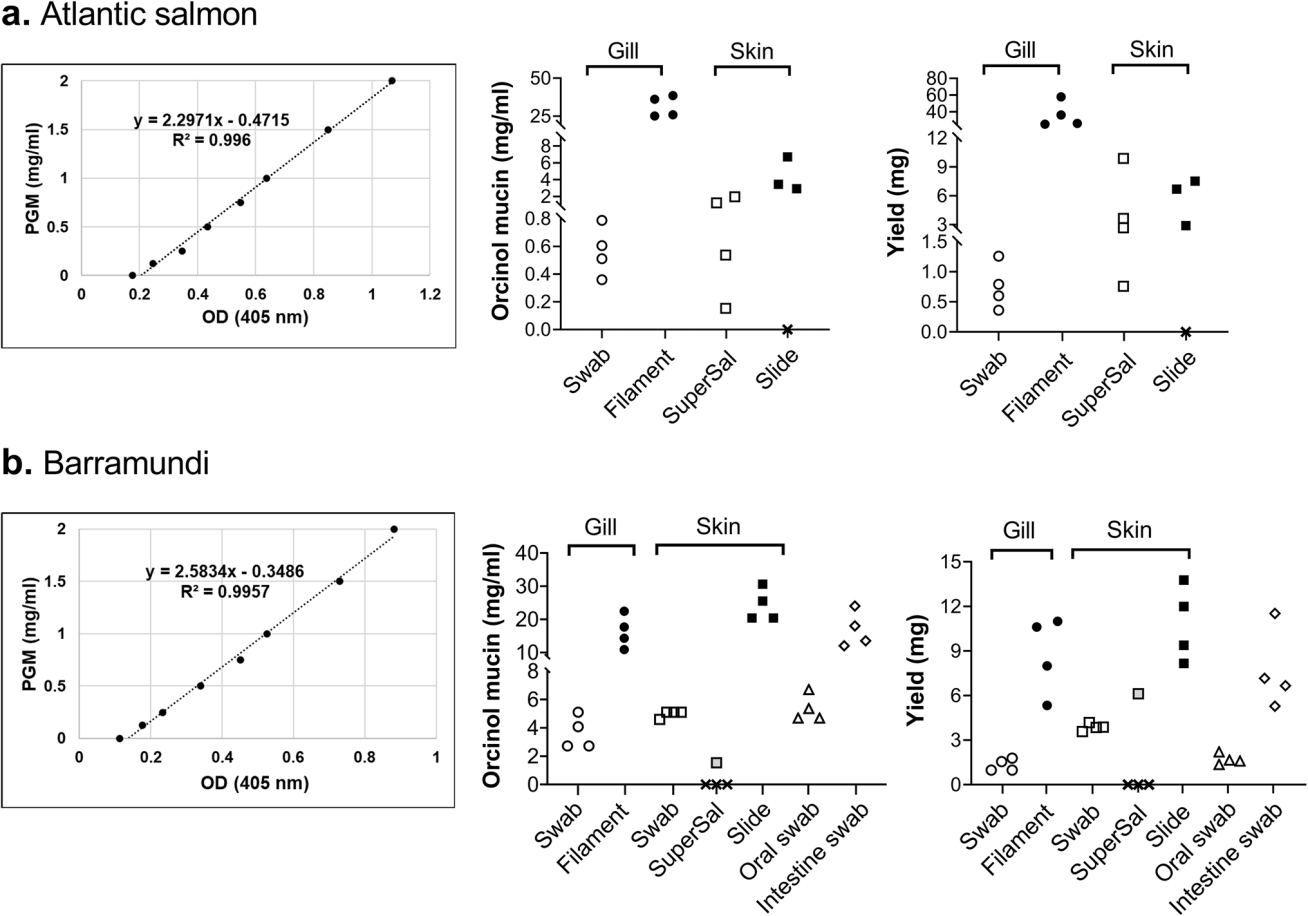
Comparison of mucin yield according to sampling methods based on the orcinol assay. **a** Standard curve based on PGM (left), concentration of Atlantic salmon mucin (middle) and total yield of Atlantic salmon mucin (right). **b** Standard curve based on PGM (left), concentration of barramundi mucin (middle), and total yield of barramundi mucin (right). *X* symbolises that no mucin was detected using this method for this sample. All samples were run in duplicate, and the data shown in the graphs are the mean of the duplicate

Based on semi-quantification using the orcinol assay in combination with a standard curve of PGM and multiplication of the signal with a factor to compensate for the difference in hexose content between the mucins from the different epithelial sites and PGM (see Table 1), the concentration as well as the total amount of mucin obtained from the gill filament whole tissue extract was higher than that obtained from the gill swab in both fish species (Fig. 2). Super·SAL™ resulted in good concentration and yield for Atlantic salmon skin mucus (similar yield as scraping with a glass slide, ~ 2 mg), but the amount of mucus obtained from barramundi skin using Super·SAL™ was below the detection limit for three out of four samples (Fig. 2). For barramundi skin, the glass slide resulted in three-fold higher yields than the swab; however, the ~ 4 mg obtained by the swab is a sufficient yield for many assays. Mucins were successfully detected in all samples from the oral cavity and intestine collected by swab (Fig. 2b).

**Fig. 3:**
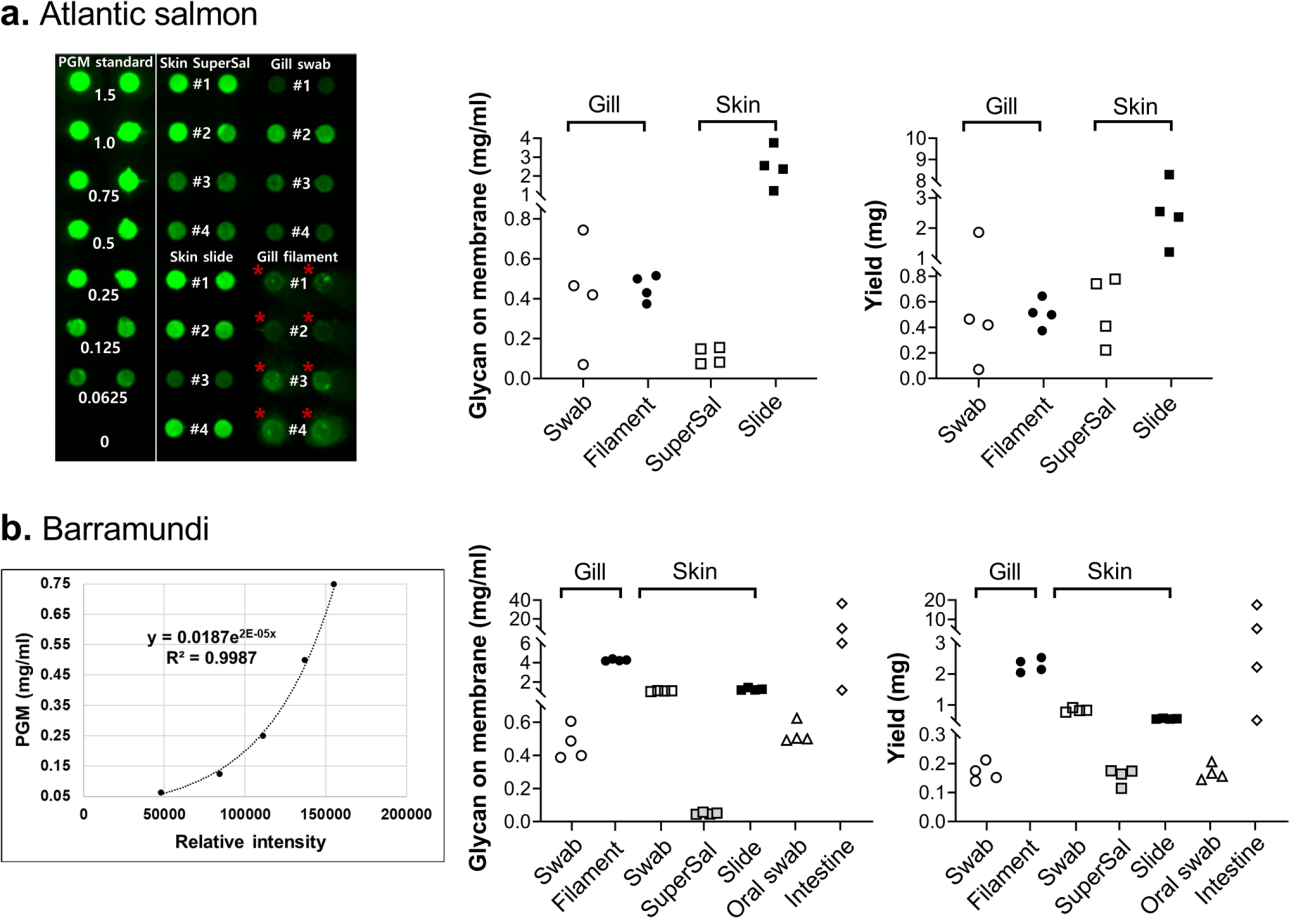
Comparison of mucin yield according to sampling methods based on the glycan-on-membrane assay. **a** Atlantic salmon: example of a glycan-on-membrane assay, included to visualise how sample overloading can be identified on the membrane, as can be seen for the gill filament samples, marked as red asterisks. The dots labelled as #1, #2, #3, and #4 represent four fish per each sampling method group (left). Concentration of Atlantic salmon mucin (middle) and total yield of Atlantic salmon mucin (right). **b** Barramundi: Standard curve based on PGM (left), concentration of barramundi mucin (middle), and total yield of barramundi mucin (right). All samples were run in duplicate, and the data shown in the graphs are the mean of the duplicate

The semi-quantification of mucin concentration and yield based on the glycan-on-membrane assay gave relatively similar results to the orcinol-based assay, with two exceptions: Firstly, a signal higher than the background signal was obtained for all samples, i.e. the sensitivity of the glycan-on-membrane assay was higher than the orcinol-based assay (Fig. 3). Secondly, the Atlantic salmon gill whole filament samples were very slow to pass through the membrane and spread outside the dots in the dot-blot manifold (Fig. 3a, marked as red asterisks). This issue can be solved by diluting the sample further, however, we show this dilution here to highlight the drawback that samples can clog the membrane in the glycan-on-membrane assay if not diluted sufficiently. Note that the results from the two assays were not sufficiently similar to use the assays interchangeably, e.g. careful consideration should be given to which of the methods better reflects the use of the mucus in the downstream assays planned: if the terminal epitopes are the important ones, it is most useful to use the glycan-on-membrane assay, whereas if the total number of monosaccharides is important, it is suitable to use the orcinol assay.

Overall, the glycan-on-membrane assay likely results in more accurate mucin estimation and appears more convenient than the orcinol-based assay for most applications, due to the fact that i) potential *N*-glycan content/contamination can have a disproportionally large impact on the orcinol-based assay signal, especially among mucins with a very low non-amine hexose content, ii) it is a major complication to have to characterise the mucin glycan repertoire for species with unknown *O*-glycomes due to the expense and significant amount of sample and human resources required. Furthermore, the glycan-on-membrane assay will give a rough estimation of the concentration even without the use of a compensation factor, as the factor is approximately one for all samples investigated so far, iii) the accuracy in concentration is likely affected among samples that have a notable difference in non-amine hexose content compared to the sample used for setting the standard curve for the orcinol assay. iv) It is better to have an assay that measures the majority of the glycans, as relatively small structural differences between samples can have a large impact otherwise. The glycan-on-membrane assay elicits a signal based on the terminal epitopes, which is often the part of the molecule of interest, for example in interactions with pathogens, and v) the inter-individual variation in glycan composition had a larger impact on the signal obtained with the orcinol assay than on the signal obtained with the glycan-on-membrane assay. Furthermore, there are also reports suggesting that fucose exhibits poor sensitivity with the orcinol assay (Irwin and Leaver 1956; Graham and Neuberger 1968), and although the fucose content in the currently investigated species/sample sets was low, this factor may further complicate the interpretation of orcinol assay results for sample sets with high fucose content. However, disadvantages of the glycan-on-membrane assay are that i) the sample needs to be titrated to obtain good results (too high concentration leads to some of the sample not attaching to the membrane/spreading outside the well of the dot-blot manifold) and ii) a dot-blot manifold is needed, which is not standard equipment in many laboratories.

### Absorbance at 230 nm Estimates a Higher Mucin Concentration in the Mucus Extracts than the Established Glycan-Specific Methods, Although for Purified Mucins the Results are Similar

**Fig. 4:**
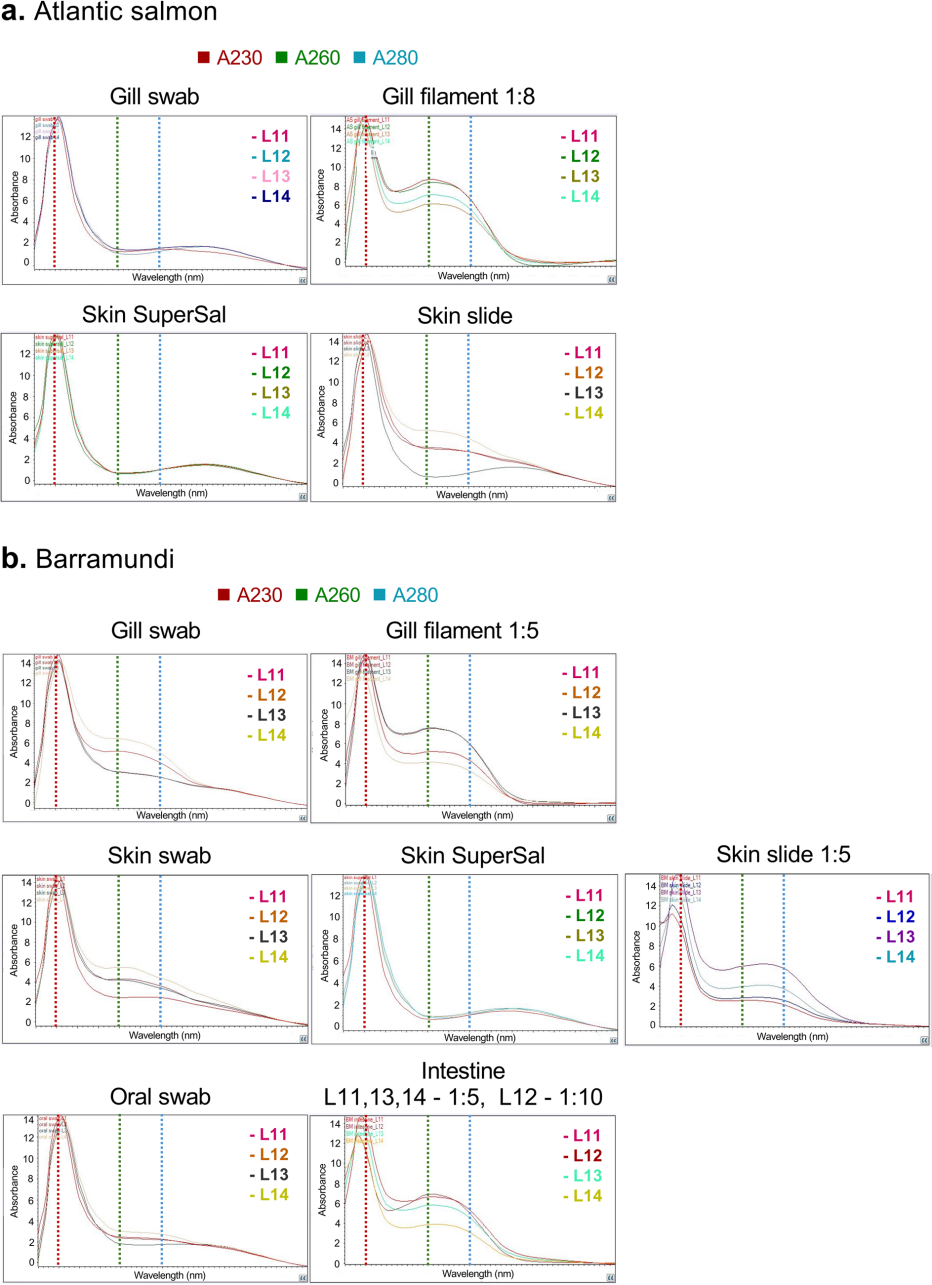
Wavelength scan (220–350 nm) of fish mucus harvested using different sampling methods. **a** Atlantic salmon; **b** Barramundi. Due to differences in the relation between the A230 peak and the broad curve present at higher wavelengths between samples at different concentrations (see Fig. 5), samples are shown either at their original concentration after extraction or further diluted so that all samples have an A230 of approximately 14 to allow for comparisons between the curves. For the intestinal samples, L11–14 denotes different samples that required to be diluted either 1:5 or 1:10 to achieve the absorbance at 230 nm required

**Fig. 5:**
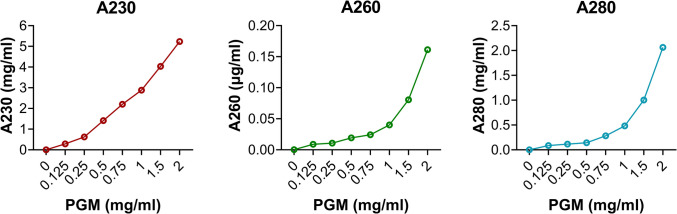
Relation between PGM concentrations and A230, A260, and A280

All mucus extracts from Atlantic salmon (Fig. 4a) and barramundi (Fig. 4b) displayed a pronounced peak at 230 nm followed by a flatter curve. For samples collected by swab, slide or Super·SAL™, the second curve was relatively small (5–40% of the height of the peak at 230 nm), whereas for the gill filament homogenate extracts, which contain also a high proportion of non-mucus material, this peak was approximately 50–70% of the height of the peak at 230 nm. Notably, the relation between the A230, A260 and A280 changed with the dilution of the sample. Examples of this are presented for PGM (Fig. 5). This appears to be due to the fact that the A230 peak had a linear relationship with the concentration, whereas the A260 and A280 had an exponential relationship with the concentration of the sample (Fig. 5). Therefore, it appears important to compare the shape of the scans from samples at approximately the same concentration if using this as an estimate of mucus purity.

**Fig. 6:**
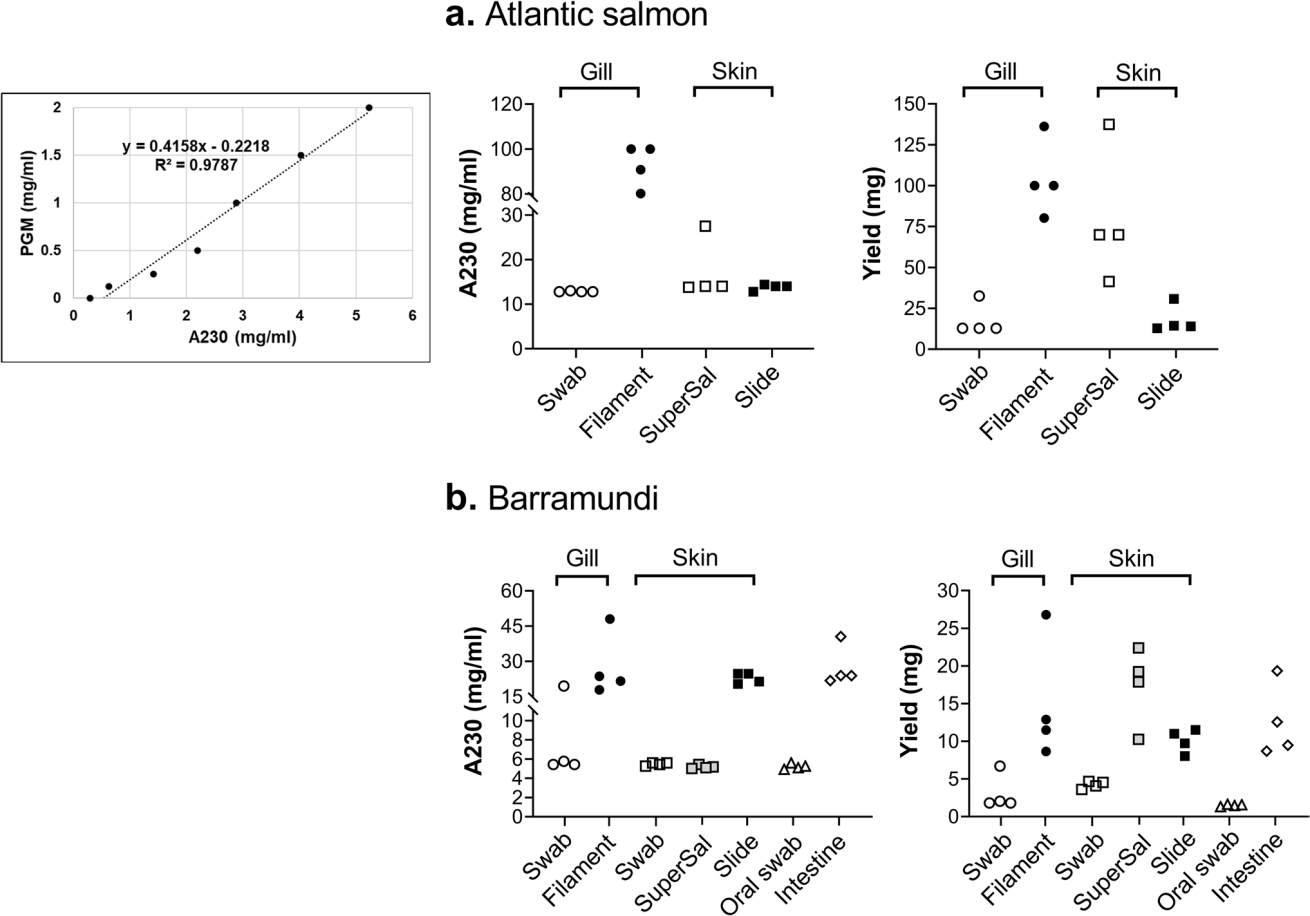
Comparison of mucin yield according to sampling methods based on absorbance at A230 nm. Left: standard curve based on PGM. **a** Concentration of Atlantic salmon mucin (middle) and total yield of Atlantic salmon mucin (right). **b** Concentration of barramundi mucin (middle) and total yield of barramundi mucin (right)

Absorbance measurements are very fast and can be useful to determine the concentration of biomolecules. For example, protein concentration is often estimated by measuring A280 and DNA concentration by measuring A260. Measuring A280 to determine the mucin concentration based on its protein content is suboptimal, as the overlap with the absorbance signal for DNA (a common contaminant in mucus extracts) at 260 nm is significant and mucins elicit a relatively low signal in protein analyses due to the fact that the majority of the molecule is composed of carbohydrate. Therefore, the main peak from the mucus extracts at A230 was used as a measure together with a standard curve based on PGM. However, although the samples that indicated a high concentration based on the orcinol and glycan-on-membrane assays also indicated a high concentration using the A230 measurement, the A230 measurement indicated notably higher (7- to 200-fold) concentrations than the established orcinol and glycan-on-membrane assays (compare Figs. 2, 3 and 6). Since several molecules in addition to carbohydrates are known to absorb at A230 and these samples are mucus extracts, not pure mucins, results obtained by the glycan-on-membrane assay are more reliable than A230. The notion that non-mucin molecules markedly contributed to the A230 signal of the mucus extracts is further supported by the fact that the A230 and glycan-on-membrane assays yielded similar results for mucins purified by isopycnic density gradient from Atlantic salmon gill (0.71 versus 0.80 mg/mL, respectively) and intestine (0.52 versus 0.53 mg/mL, respectively).

### Methodological Considerations

Quantifying mucin is difficult and the method used impacts the results, so it is necessary to understand the characteristics of the semi-quantitative methods and select the appropriate method based on the purpose of the experiment. In addition to the methods used in this study, which either result in a signal proportional to the number of non-amine hexoses in the glycan structure (the orcinol assay) or mainly reflects the number of “branches” on the glycan (the glycan-on-membrane assay), there are also Alcian blue-based assays (which reflect) the degree of sialylation and sulfation of the glycans) and a 2-cyanoacetamide-based assay (which reflects the number of glycan chains without taking the glycan size or number of “branches” into account) (Crowther and Wetmore 1987; Padra and Lindén 2021). For studies focused on mucin pathogen binding ability, the glycan branches are often important and it might then be useful to use the glycan-on-membrane assay, although the 2-cyanoacetamide-based assay is also a valid choice, while studies on bacterial utilisation of glycans as a nutrient source might deem the glycan size an important factor, thereby making the orcinol assay a suitable choice.

Note that commercially available mucins used for standard curves can vary between batches and may contain various contaminants. Therefore, the methods described here are useful for providing an estimation of the concentration in samples within experiments to determine if there are differences in mucin concentration between large groups of fish that have received different treatments, or to set the concentration in samples so they are similar for downstream analysis.

None of the four methods listed above generates an exact concentration, nor do they take the purity of the sample into account. The wavelength scan used here was rapid and therefore easily applied to large sample sets to visualise the presence of large amounts of non-mucin material in samples. However, moderate levels of contamination are not so easily detected. Possible ways of detecting contaminants include mixed sample assays for protein (which is relatively low in proportion in mucins) or DNA (not part of the mucin), as well as chemical detection of samples run on sodium dodecyl sulfate-agarose/polyacrylamide composite gels (Schulz et al. 2002). However, it is a challenge to locate assays that are not sensitive to the large concentration of chaotropic agent often used in the mucin extraction process, and the GuHCl used in the current study is an issue for many assays as well as for electrophoresis, making it necessary to dialyse samples before such analysis.

## Conclusion

Overall, we conclude that swabs were the most versatile tool for sampling mucus, allowing for sufficient amounts of mucus to be harvested with relative ease and low levels of contamination (e.g. blood) from the oral cavity, gill, skin, and intestine. The molecular basis for eliciting a signal was much more similar between fish epithelia for the glycan-on-membrane method than for the orcinol assay, and determining concentration based on A230 nm absorbance was only useful for purified mucin samples, not mixed samples. Therefore, we recommend the glycan-on-membrane assay over the other two methods for samples containing a mixture of molecules. We further recommend using at least two dilutions for each sample, as the signal can reach a plateau. All mucus extracts displayed a pronounced peak at 230 nm followed by a flatter curve, and the relation between the peak at 230 nm and the flatter curve at higher wavelengths gave an indication of the purity of the sample. Notably, the relation between the A230, A260, and A280 changed with the dilution of the sample, because the A230 peak has a linear relation with the concentration, whereas the A260 and A280 have an exponential relation with the concentration of the sample. Therefore, it is useful to estimate both sample concentration and purity by comparing samples at approximately the same concentration.

## Data Availability

The glycans from barramundi and Atlantic salmon can be found at https://unicarb-dr.glycosmos.org/references/GPST000507.0/f_0000045258, https://unicarb-dr.glycosmos.org/references/392, and https://doi.org/10.1021/acs.jproteome.5b00232. The remainder of the data are shown in the figures.
